# Substrates and Inhibitors of SAMHD1

**DOI:** 10.1371/journal.pone.0169052

**Published:** 2017-01-03

**Authors:** Joseph A. Hollenbaugh, Jadd Shelton, Sijia Tao, Sheida Amiralaei, Peng Liu, Xiao Lu, Russell W. Goetze, Longhu Zhou, James H. Nettles, Raymond F. Schinazi, Baek Kim

**Affiliations:** 1 Center for AIDS Research, Laboratory of Biochemical Pharmacology, Department of Pediatrics, Emory University School of Medicine, Atlanta, Georgia United States of America; 2 Department of Biomedical Informatics and Department of Pediatrics, Emory University School of Medicine, Atlanta, Georgia United States of America; 3 Children’s Healthcare of Atlanta, Atlanta, Georgia United States of America; 4 College of Pharmacy, Kyung-Hee University, Seoul, South Korea; Meharry Medical College, UNITED STATES

## Abstract

SAMHD1 hydrolyzes 2'-deoxynucleoside-5'-triphosphates (dNTPs) into 2'-deoxynucleosides and inorganic triphosphate products. In this paper, we evaluated the impact of 2' sugar moiety substitution for different nucleotides on being substrates for SAMHD1 and mechanisms of actions for the results. We found that dNTPs ((2'*R*)-2'-H) are only permissive in the catalytic site of SAMHD1 due to L150 exclusion of (2'*R*)-2'-F and (2'*R*)-2'-OH nucleotides. However, arabinose ((2'*S*)-2'-OH) nucleoside-5'-triphosphates analogs are permissive to bind in the catalytic site and be hydrolyzed by SAMHD1. Moreover, when the (2'*S*)-2' sugar moiety is increased to a (2'*S*)-2'-methyl as with the SMDU-TP analog, we detect inhibition of SAMHD1’s dNTPase activity. Our computational modeling suggests that (2'*S*)-2'-methyl sugar moiety clashing with the Y374 of SAMHD1. We speculate that SMDU-TP mechanism of action requires that the analog first docks in the catalytic pocket of SAMHD1 but prevents the A351-V378 helix conformational change from being completed, which is needed before hydrolysis can occur. Collectively we have identified stereoselective 2' substitutions that reveal nucleotide substrate specificity for SAMHD1, and a novel inhibitory mechanism for the dNTPase activity of SAMHD1. Importantly, our data is beneficial for understanding if FDA-approved antiviral and anticancer nucleosides are hydrolyzed by SAMHD1 *in vivo*.

## Introduction

Sterile alpha motif (SAM) domain and histidine/aspartic acid (HD) domain containing protein 1 (SAMHD1) hydrolyzes canonical 2'-deoxynucleoside-5'-triphosphates (dNTPs) into deoxynucleosides and inorganic triphosphates (PPP) products [1, 2]. The dNTP triphosphohydrolase activity occurs in the HD domain of SAMHD1. The dNTPase activity appears to require homotetramer complex assembly, which is regulated by sequential binding of allosteric activators [3, 4]. However the SAMDH1C (120–626) truncated protein, which cannot form homotetramers, has been shown to have dNTPase activity [1, 5]. Further studies are needed to determine if putative interacting protein partners, Aicardi–Goutières syndrome mutations, other point mutations or other truncation mutants of SAMHD1 may block homotetramerization while still permit dNTPase activity [1, 5–8]

It has been shown that SAMHD1 inhibits retrovirus infection, most likely by acting as a key regulator for cellular dNTPs levels, which can influence such infection [9–11]. Recent reports indicate that T592 phosphorylation of SAMHD1 also influences retrovirus restriction in myeloid cells and can modulate triphosphohydrolase activity [12–15]. Furthermore, SAMHD1 has been investigated in the context of how cellular dNTP concentrations influence the efficacy of nucleoside reverse transcriptase inhibitors (NRTIs) for lentiviral infection [16–19]. Nucleoside derivatives–ribonucleoside, 2'-deoxyribonucleoside or arabinose nucleoside analogs–are antimetabolites, which represent an important class of chemotherapeutic agents used to treat cancers and viral infections [20–22]. These antimetabolites enter the cell through active transport mechanisms and require phosphorylation by several cellular kinases to produce their monophosphate (MP), diphosphate (DP) and triphosphate (TP) analog forms. In addition, certain 2'-deoxynucleoside-5'-TP analogs, arabinose nucleoside-5'-TP analogs and dUTP can compete with naturally occurring canonical dNTPs as substrates for host DNA polymerases or viral DNA polymerases [23, 24]. This competition promotes mutagenesis and apoptosis of cancer cells or termination of viral replication [25, 26]. Therefore, maintaining a proper cellular dNTP balance is important biologically for ensuring DNA fidelity during replication and repair [27–30].

In this study we specifically explore the effect of stereoselective 2' substitution of nucleoside analogs on SAMHD1 activity. We use computational modeling to place our results into a structural context that may help better understand the larger mechanistic details of SAMHD1 substrate specificity. The L150 and Y374 of SAMHD1 have been proposed to contribute to the formation of a tight catalytic pocket [31]. Using an HPLC-based assay, we found that the L150 of SAMHD1 acted as a steric gate to prevent (2'*R*)-2'-F-dCTP and CTP from being hydrolyzed by SAMHD1, which supports published results [31]. Modeling of the catalytic site with dCTP and ara-CTP ((2'*S*)-2'-OH) did not show a clash with Y374, whereas SMDU-TP analog did show a clash. Biochemical analysis showed that dCTP and ara-CTP were substrates for SAMHD1, whereas SMDU-TP blocked dNTPase activity of SAMHD1. Y374 is part of a helix that undergoes a conformational change to move closer to the dNTP [5]. We hypothesize that the (2'*S*)-2'-methyl substituted nucleotide (SMDU-TP) blocks the helix conformation change at the catalytic site, providing a novel mechanism for inhibiting the dNTPase activity of SAMHD1. Collectively, our data show that stereoselective 2' substitution for nucleotides can impact the substrate specificity and dNTPase activity of SAMHD1.

## Materials and Methods

### Compounds

Gemcitabine (2',2'-diF-dC), arabinose-C (ara-C; aka cytarabine), dCMP were purchased from Sigma. Gemcitabine-5'-triphosphate (gem-TP), ara-cytidine-5'-triphosphate (ara-CTP) and 5-aza-2'-deoxycytidine-5'-triphosphate (decitabine-TP) were purchased from Jena Bioscience. dGTP, dATP and dCTP was purchased from Affymetrix. ATP, CTP and GTP were purchased from Thermo Scientific. 2',3'-dideoxyinosine-5'-triphosphate (ddITP), 2',3'-dideoxadnosine-5'-triphosphate (ddATP), 2',3'-dideoxcytosine-5'-triphosphate (ddCTP) and 2',3'-dideoxguanosine-5'-triphosphate (ddGTP) were purchased from Roche. Cytarabine-^13^C_3_ was purchased from Toronto Research Chemicals. (2'*R*)-2'-F-2'-deoxyadenosine-5'-triphosphate (2'-F-dATP) and (2'*R*)-2'-F-2'-deoxycytidine-5'-triphosphate (2'-F-dCTP) were purchased from TriLink BioTechnologies.

### Recombinant SAMHD1-GST purification

Human SAMHD1 was cloned into pGEX-6P-1 with an N-terminal GST tag (GE Healthcare, provided by Dr. Yoshio Koyanagi) and transformed into BL21 (DE3) pLysS competent cells (Invitrogen). Cells were grown at 37°C to an A600 of 0.5, stored on ice for 2 h, and induced overnight with 0.25 mM isopropyl-α-D-1-thiogalactopyranoside at 25°C. Cells were harvested and lysed in lysis buffer (50 mM Tris-HCl [pH 7.5], 500 mM NaCl, 2 mM EDTA, 1 mg/ml chicken egg white lysozyme, and one tablet of Roche Applied Science Complete protease inhibitor mixture) for 4 h on ice. Cell debris was removed by centrifugation at 49,000 × *g* for 15 min, and lysate was incubated overnight at 4°C with 1.5 mL of Glutathione Sepharose^®^ 4B bead slurry (GE Healthcare). Beads were pelleted and washed three times with wash buffer (50 mM Tris-HCl [pH 7.5], 500 mM NaCl, 1 mM dithiothreitol, 0.5% Triton X-100), equilibrated in buffer (50 mM Tris-HCl [pH 7.5], 150 mM NaCl, 20% glycerol, 0.5% Triton X-100) and packed into a column. The column was washed three times with 30 mL of equilibration buffer, and SAMHD1 was eluted with 50 mM Tris-HCl [pH 8], 1 mM EDTA, 10% glycerol, 300 mM NaCl, 200 mM reduced glutathione. A Millipore Centricon protein concentrator (45 MWCO) was used to concentrate the protein and for buffer exchanges. Protein samples were snap frozen using liquid nitrogen and stored at -80°C until use.

### HPLC-based SAMHD1 Phosphohydrolase Assay

To measure dNTP triphosphohydrolase activity of SAMHD1, 1.6 μM recombinant SAMHD1-GST (SAMHD1) was incubated with different 500 μM nucleoside-5'-triphosphate substrates in the presence of 500 μM dCMP, 500 μM GTP and reaction buffer (50 mM Tris-HCl [pH 8], 100 mM KCl, 5 mM MgCl_2_, and 0.1% Triton X-100). Reactions were incubated for 2 h at 37°C and terminated by incubation for 10 min at 75°C. Reactions were separated and quantified by anion exchange HPLC method [32]. Separation was done using two DNAPac PA100 columns equilibrated with running buffer (25 mM Tris–HCl [pH 8] and 0.5% acetonitrile) for 10 min, 30 μL sample was injected and eluted with a linear gradient of 240 mM NH_4_Cl for 12 min, run at an isocratic gradient with 240 mM NH_4_Cl for 5 min, and column was again equilibrated with running buffer (Beckman Coulter System Gold 126 Solvent Module). Absorbance was measured with a Beckman Coulter System Gold 166 Detector at 254 nm. The amounts of deoxycytidine-5'-monophosphate (dCMP), dGTP and (deoxy)nucleoside-5'-TP analogs were determined by integrating the peak area using 32 Karat 8.0 Software. Data was normalized to dCMP peak area for each sample, used as a sample loading control. Determining changes for different (deoxy)nucleoside-5'-triphosphates of interest was calculated by setting sample without SAMHD1 peak area to 100%.

### Cells and cell culture

Monocytes were isolated from whole blood (New York Blood Service, Long Island New York) by using MACS^®^ CD14^+^ beads as described previously [33] and cultured in the presence of 5 ng/mL human GM-CSF (Miltenyi Biotec). MDMs were utilized at day 7 of maturation for experiments.

### Virus-like particles generation (VLP)

T225 flasks containing 293FT cells (Invitrogen) were transfected with 40 μg of pSIV 3+ with or without Vpx (Vpx+ VLP and Vpx- VLP, respectively; kindly provided by Dr. Nathaniel Landau) and 20 μg of pVSV-G at a ratio of 1 μg of DNA to 3 μL of polyethylenimine linear MW 25,000 (Polysciences Inc.). The following day, medium was replaced with fresh DMEM medium containing 5% FBS and antibiotics. On days 2–3 after transfection, the medium was collected and replaced with fresh medium. On the day of collection, medium was centrifuged at 400 x *g* for 5 min to remove cells. Supernatant was overlaid on top of 5 ml of a 25% sucrose cushion (25% (w/v) sucrose, 10 mM Tris-HCl [pH 7.5], 0.1 M NaCl and 1 mM EDTA). VLP were concentrated at 82520 x *g* in an SW32 Ti rotor for 90 min by ultracentrifugation. Supernatant was aspirated, and pellets were suspended in 600 μL of serum-free DMEM. Supernatant was centrifuged for 1 min at 20800 x *g* to remove debris using a tabletop centrifuge. Aliquots (50 μL) were stored at -80°C. The p27 antigen level was determined using an ELISA kit (Advanced BioScience Laboratories, Inc.). A minimum of 145 ng of p27/million cells was used.

### HLPC-MS/MS quantification of dNTPs and NTPs

The HPLC system was a Dionex Packing Ultimate 3000 modular LC system comprising of a ternary pump, vacuum degasser, thermostated autosampler, and thermostated column compartment (Dionex, CA). A TSQ Quantum Ultra triple quadrupole mass spectrometer (Thermo Scientific, Waltham, MA, USA.) was used for detection. Thermo Xcalibur software version 2.0 was used to operate HPLC, the mass spectrometer and to perform data analyses. Gradient separation was performed on a Hypersil GOLD column (100 x 1 mm, 3 μm particle size; Thermo Scientific, Waltham, MA, USA). Mobile phase A consisted of 2 mM ammonium phosphate and 3 mM hexylamine. Acetonitrile was increased from 8 to 40% in 10 min, and kept at 40% for 2 min. Equilibration at 8% acetonitrile lasted 15 min. The total run time was 27 min. The flow rate was maintained at 50 μL/min and a 25 μL injection was used. The autosampler and the column compartment were maintained at 4.5 and 30°C, respectively. Calibration curves were generated using gem-TP, and ara-CTP to determine concentrations.

### Compound synthesis

The protocol published by Seamon *et al*. was used for the synthesis of 5'-methylene-2'-deoxyuridine-5'-triphosphate (pppCH_2_dU) [34]. An anomeric mixture (10:1 ß:α) of (2'*S*)-2'-*C*-Me-2'-deoxyuridine (SMDU) [35] was synthesized using the procedure by Li and Piccirilli [36]. Chromatographic separation of the pure beta anomer was subsequently performed using a SorbTech Sorbet Technologies column on a Combiflash Teledyne Isco chromatography machine. PSI-6206 was synthesized as reported in [37]. Finally, the triphosphate forms of SMDU and PSI-6206 were prepared with >95% purity following a nucleoside derivative triphosphate synthesis procedure reported by Zhou *et al*. [38], generating SMDU-TP and PSI-6206-TP compounds.

### TLC-based SAMHD1 Phosphohydrolase Assay

SAMHD1 (1 μM) was incubated with 1.25 μCi/μL [γ-^32^P]-dTTP and various concentrations: 3,000, 1,000, 300, 100 and 30 μM of unlabeled dTTP, 500 μM GTP (as the activator), and various concentrations: 1000, 300, 100, and 30 μM of either SMDU-TP or pppCH_2_dU in reaction buffer (50 mM Tris-HCl [pH 8], 100 mM KCl, 5 mM MgCl_2_, and 0.1% Triton X-100). Reaction volume (10 μL) was incubated for 20, 40, 60, 90, 120, 180, and 300 s at 20°C. One microliter was removed from the reaction at the indicated times and stopped in 5 μL of 500 mM EDTA that was on wet ice. Samples were then heat inactivated at 95°C for 2 min before being stored at 4°C. Cellulose 300 PEI/UV_254_ TLC plates (Macherey-Nagel; Cat # 801063) were prepared by spraying with 100% methanol and then allowed to dry. Plates were marked with a pencil one inch from the top and bottom on the plate. One microliter of the reaction was spotted one inch from the bottom on TLC plates. Solvent (0.8 M LiCl, 0.05 M EDTA and 1 M acetic acid) front was allowed to migrate to within one inch from the top of the plate before the plate was removed and dried. TLC plates were exposed to Bio-Rad phosphoimager screen. Data was captured using PharosFX Plus Imager. Data was quantitated using Quantity One software (Bio-Rad).

### Western blot analysis

Samples were processed in radioimmunoprecipitation assay buffer containing 1 μM DTT, 10 μM PMSF, 10 μL/mL phosphatase inhibitor (Sigma), and 10 μL/mL protease inhibitor (Sigma). The cells were sonicated with three 5-sec pulses to ensure compete lysis. Cellular debris was removed by centrifugation at 23000 x *g* for 10 min. Supernatants were stored at -80°C before use. Cell lysates (25 μg) were resolved on 8% SDS-PAGE. Proteins were transferred to a nitrocellulose membrane. The membrane was blocked with 2% nonfat milk in TBST (10 mM Tris, 150 mM NaCl, and 0.1% Tween 20) for 1 h followed by the addition of primary antibodies: SAMHD1 (Abcam), and GAPDH (Santa Cruz). Cut membrane was incubated overnight with antibodies at 4°C. The next day, the membrane was washed (3x, 20 min with TBST) and treated with anti-mouse-HRP or anti-rabbit-HRP (GE Healthcare) for 1 h at room temperature. Membrane was washed (3x, 20 min with TBST) and developed using the SuperSignal West Femto Kit (Thermo Scientific). Images were captured using a Bio-Rad ChemiDoc Imager. ImageLab Analysis software (Bio-Rad) was used to analyze the data.

### Graphing and statistical analysis

Prism (GraphPad) software was used for plotting the data. Graphs are plotted as the means and standard error of means (SEM). All the data sets were compared for significant difference using Two-way ANOVA analysis and either Bonferroni post-test analysis for significance with the dNTP data or multiple comparisons. The *Km* and *Ki* values for dTTP and SMDU-TP are determined using Prism software.

## Results

### Cartoon for SAMHD1 homotetramerization

**Fig 1:**
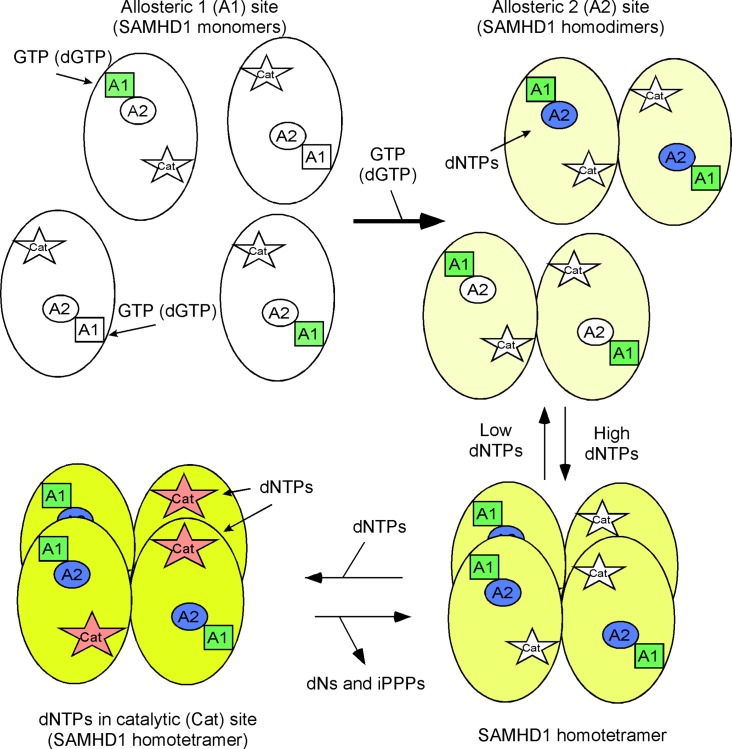
Cartoon for SAMHD1 homotetramerization. SAMHD1 monomers bind GTP (or dGTP) at allosteric 1 (A1) site leading to the formation of homodimers. Next, dNTPs bind to allosteric 2 (A2) sites allowing for the formation of homotetramers. The catalytic (Cat) site then can accompany dNTPs for hydrolysis, leading to the generation of deoxynucleosides (dNs) and inorganic triphosphates (iPPP).

The process of SAMHD1 tetramerization begins when SAMHD1 monomers binds GTP or dGTP at the allosteric 1 (A1) site to promote homodimer formation (Fig 1) [39, 40]. The intracellular GTP concentration is maintained at around 400 μM as compared to 1–3 μM dGTP in activated T cells and 40 nM dGTP in macrophages [41, 42]. At these physiological levels, GTP should always be docked within the A1 site and limit the amount of free SAMHD1 monomer within the cell [40]. The SAMHD1 homodimers then bind canonical dNTPs at allosteric 2 (A2) sites, allowing for a homotetramer complex to form. The homotetramer of SAMHD1 then permits binding of dNTPs into the catalytic (Cat) sites. Some of the interactions know are metal ion, coordinated by H167, H206, D207 and D311, binds to the γ-phosphate to further stabilize the dNTP in the catalytic pocket of SAMHD1. There are interactions with the different bases that contribute to *Km* differences between the different dNTPs. Initiating dNTP triphosphohydrolase activity appears to occur after the dNTP has docked in the catalytic site of SAMHD1 and conformational change in the homotetramer [39]. Comparison of dGTP-bound tetramer to non-substrate bound dimer (3U1N) showed that the A351-V378 helix moves closer to the dNTP allowing for better substrate binding within the binding pocket [5]. Once the helix conformational change has been completed, then R366 may interact with the γ-phosphate to further stabilize the dNTP in the catalytic pocket. Hydrolysis occurs by the attack of the phosphodiester bond between the α-phosphate and sugar to liberate dN and iPPP products from the four active sites of SAMHD1 [5, 31]. Several laboratories have characterized and reported substrate specificity of SAMHD1 for ribonucleoside-5'-triphospahte (rNTPs), dNTPs and HIV nucleoside reverse transcriptase inhibitors [1, 16–18, 34, 39, 40, 43]. It should be noted that all the canonical dNTPs are substrates for SAMHD1 as well as competitive inhibitors amongst themselves due to their slight differences in *Km* values [44].

### Role of the 2'*R* and 3'*R* sugar moieties for nucleotide hydrolysis

**Fig 2:**
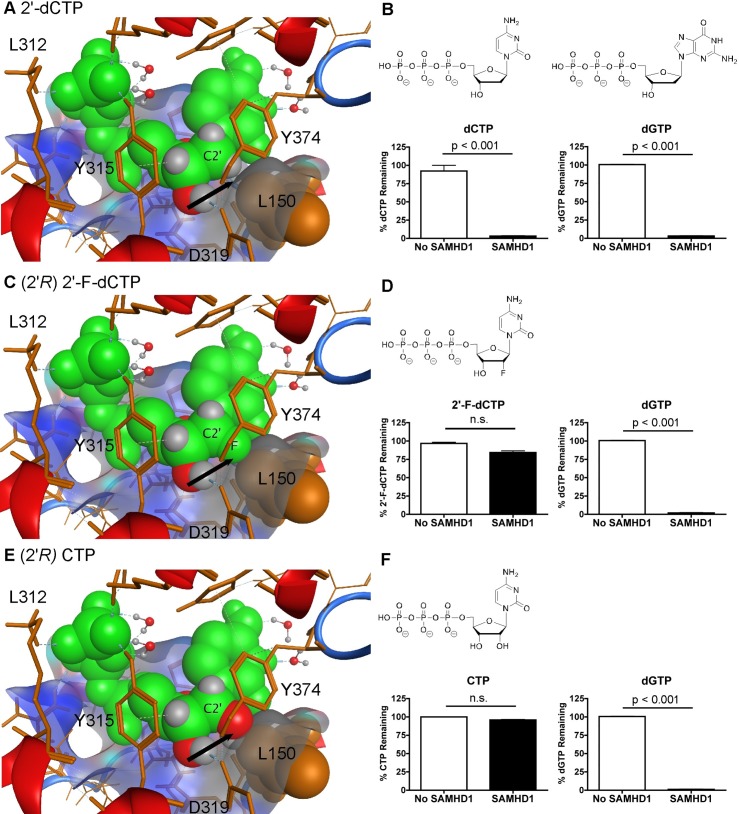
Examining the role of L150 for nucleotide specificity. A) dCTP, C) (2'*R*) 2'-F-dCTP and E) CTP nucleotides (in green) are modeled in the catalytic site of SAMHD1. L150 clashes with (2'*R*) 2'-F-dCTP and CTP, but not dCTP (see arrows) within the catalytic pocket of SAMHD1. B, D and F) Determining if dCTP, (2'*R*) 2'-F-dCTP and CTP can be hydrolyzed for SAMHD1 *in vitro*. Structures of the compounds are above the HLPC graphs. Using semi-quantitative HLPC analysis method, compounds were incubated with and without 1.6 μM of SAMHD1 enzyme plus dGTP (A1 site activator) to determine if they are substrates of SAMHD1. Data are presented as the percent compound remaining (y-axis). dCTP and dGTP were significantly hydrolyzed (p < 0.001; T test). No significant (n.s.) differences were detected between samples with and without SAMHD1 protein for (2'*R*) 2'-F-dCTP and CTP analogs. HPLC analysis of each nucleoside was done twice in triplicate. Mean and SEM are plotted with significant or no significant (n.s.) differences determined by T test analysis.

Ji *et al*. reported the crystal structure of SAMHD1 and suggested that L150 and Y374 were involved in generating a tight catalytic binding pocket [31]. These amino acids might exclude rNTPs, which has a (2'*R*)-2'-OH sugar moiety (ribose), from docking at the catalytic pocket due to a steric clash with L150. To begin, we modeled dCTP (Fig 2A) into the catalytic site of SAMHD1 with the point of view focused on the L150 of SAMHD1. We observed that dCTP fits within the catalytic site of SAMHD1 without touching L150, see arrow (Fig 2A). Next, we tested dCTP using a semi-quantitative HPLC-based assay [32]. Essentially, nucleotide analogs were incubated with and without 1.6 μM of SAMHD1 enzyme and dGTP, which acts as the A1 site activator and also an internal positive control to ensure the enzyme is working. HPLC data were analyzed by calculating the changes in peak area of the compound for with and without SAMHD1 protein, while using dCMP as an internal loading control. The normalized peak area for the SAMHD1 negative control (no SAMHD1) reaction was set to 100% analog remaining. Data for reactions containing SAMHD1 protein are then statically compared to the no SAMHD1 reactions (*n* = 3). As displayed in Fig 2B, both dCTP and dGTP were significantly hydrolyzed (p < 0.001; T test) in the presence of SAMHD1. Moreover, dATP, dTTP and decitabine-TP were also tested and are also substrates for SAMHD1 (S1 Fig). Next (2'*R*)-2'-F-dCTP was modeled in the catalytic site of SAMHD1 (Fig 2C); it appears to clash with L150 (see arrow). Using the biochemical assay, (2'*R*)-2'-F-dCTP was not hydrolyzed by SAMHD1 (Fig 2D), whereas the dGTP in the same reaction tube was significantly decreased (p < 0.001) by SAMHD1. In addition, (2'*R*)-2'-F-dATP was not degraded by SAMHD1 (S1 Fig). Finally, CTP was modeled in the catalytic site of SAMHD1 and also illustrates a clash with L150 (Fig 2E). CTP was not hydrolyzed by SAMHD1 *in vitro* (Fig 2F), while the dGTP internal control was significantly hydrolyzed (p < 0.001). Consistent with the above results, ATP, GTP and UTP were not substrates for SAMHD1 (S1 Fig). Collectively, these data indicate that the sugar, not the nucleoside base, plays an important role in determining substrate specificity for SAMHD1.

**Fig 3:**
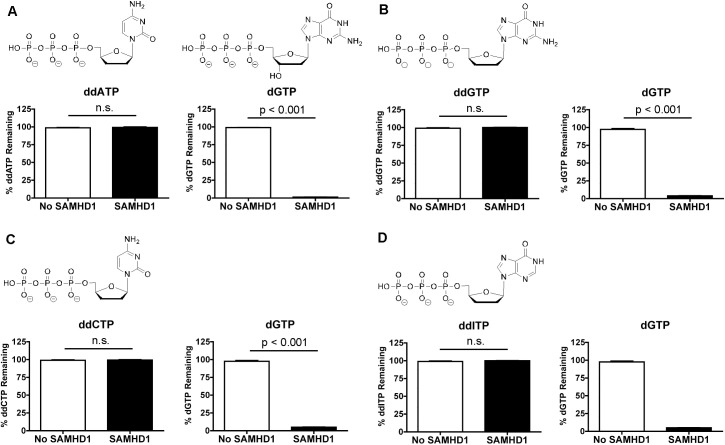
Role of 3'-OH sugar moiety for SAMHD1’s substrate specificity. A-D) Using semi-quantitative HLPC analysis method, 2',3'-ddATP, 2',3'-ddGTP, 2',3'-ddCTP and 2',3'-ddITP are incubated with and without 1.6 μM of SAMHD1 enzyme plus dGTP (A1 site activator) to determine if they are substrates of SAMHD1. Data are presented as the percent compound remaining (y-axis), showing that none of these nucleoside analogs were hydrolyzed by SAMHD1. dGTP is also used as an internal positive control and is significantly hydrolyzed (p < 0.001) by SAMHD1 in the presence of all the 2'3'-ddNTP. Mean and SEM are plotted with significant or no significant (n.s.) differences determined by T test analysis.

Ji *et al*. proposed that the 3'-OH of the sugar is required for hydrogen bonding interactions with D319 and Q149 of SAMHD1 in the catalytic pocket [31]. From Fig 2A, we illustrate D319 having a hydrogen bond interaction with the 3’-OH of dCTP. Using the biochemical assay, 2',3'-ddATP (Fig 3A), 2',3'-ddGTP (Fig 3B), 2',3'-ddCTP (Fig 3C) and 2',3'-ddITP (Fig 3D) are showed not to be significantly (n.s.) hydrolyzed by SAMHD1, nor did these analogs negatively impact dGTP hydrolysis by SAMHD1 (Fig 3A–3D). 2',3'-ddC (zalcitabine) and 2',3'-ddI (didanosine) are FDA-approved NRTI compounds to treat HIV. Our findings are consistent with previously reports [17, 40].

Collectively, these data indicate that L150 may effectively exclude any nucleotides-5'-triphosphates with a (2'*R*)-2' sugar moiety larger than a hydrogen atom from properly fitting into the catalytic site of SAMHD1. Thus L150 may act as a steric gate for SAMHD1. Secondly, the base does not restrict access to the catalytic site of SAMHD1, but it can influence the overall *Km* of the nucleotides [44], i.e., the canonical dNTPs compete between themselves at the catalytic site. Third, 3'-OH sugar moiety and being a triphosphate are essential for permitting hydrolysis of nucleotide analogs.

### Examining the role of Y374 for SAMHD1 substrate specificity

**Fig 4:**
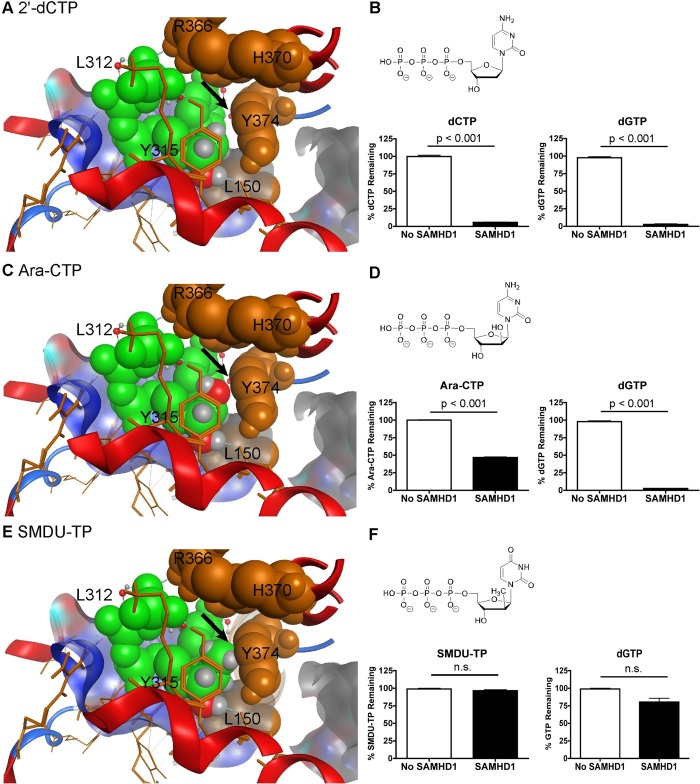
Role of Y374 and C2' sugar moiety substitution in acting as substrates of SAMHD1. A) dCTP, C) ara-CTP and E) SMDU-TP nucleotides (in green) are modeled within the catalytic site of SAMHD1. Both dCTP and ara-CTP do not clash with Y374 (see arrow). However the model shows that the (2'*S*)-2'-methyl group of SMDU-TP clashes with Y374 in the catalytic pocket of SAMHD1. B, D and F) Determining if dCTP, ara-CTP and SMDU-TP can be hydrolyzed for SAMHD1 *in vitro*. Structures of the compounds are above the HLPC graphs with experimental conditions described in Fig 2. Data are presented as the percent compound remaining (y-axis). dCTP and ara-CTP are significantly hydrolyzed (p < 0.001). SMDU-TP and dGTP, in the same reaction tube, had no significant hydrolysis in the presence of SAMHD1. HPLC analysis of each nucleoside was done twice in triplicate. Mean and SEM are plotted with significant or no significant (n.s.) differences determined using T test analysis.

We examined the contribution of Y374 within the catalytic pocket of SAMHD1 [31]. Y374 might be important for excluding (2'*S*)-2' sugar moiety modification from the catalytic site. Our modeling now focuses on the Y374 and shows that a (2'*S*)-2'-H group of dCTP (Fig 4A) fits in the catalytic site. Both dCTP and dGTP were significantly hydrolyzed (p < 0.001) by SAMHD1 (Fig 4B). We further modeled ara-CTP, which has a (2'*S*)-2'-OH group (Fig 4C). It also fits into the pocket without a clash with Y374 (Fig 4C, see arrow). We observed that both ara-CTP and dGTP are significantly hydrolyzed (p < 0.001) by SAMHD1 (Fig 4D). Additionally, we tested ara-ATP and ara-UTP, and both were degraded by SAMHD1 (S3 Fig). Finally, we examined SMDU-TP, which has a (2'*S*)-2'-methyl (CH_3_) group (Fig 4F). SMDU-TP appears to clash with Y374 of SAMHD1 (Fig 4E; see arrow). Using our biochemical assay, SMDU-TP was not hydrolyzed by SAMHD1 (Fig 3F). Moreover, dGTP hydrolysis was reduced in the presence of SMDU-TP, suggesting it may be a dNTPase inhibitor. To further investigate this analog, 0.1 mM SMDU-TP was tested in the presence of 1 mM dGTP (S3 Fig). Under these experimental conditions, dGTP was significantly hydrolyzed (p < 0.001) by SAMHD1, suggesting that SMDU-TP may act as a competitive SAMHD1 inhibitor. Additionally, PSI-6206-TP, (2'*S*)-2'-methyl, (2'*R*)-2'-F-2'-deoxyuridine-5'-triphosphate, was tested and found not to be a substrate for SAMHD1 nor did it inhibit dGTP hydrolysis (S3 Fig), which is consistent with other (2'*R*)-2'-F nucleotide analogs (Fig 2D and S1 Fig). We attempted to further evaluate if Y374 acts as a steric gate, but the Y374I, Y374F and Y374A mutants are catalytically dead (S2 Fig). Collectively, these data suggest that a (2'*S*)-2' sugar moiety as large as a methyl group is permissive for entry into the catalytic site of SAMHD1. Our model illustrations are based on crystal structures that are closed around an α-thio-dGTP, which is a poorly hydrolysable substrate [31]. Since the helix A351-V378 moves 10Å towards the dNTP in order to bind the substrate better [5], we speculated that SMDU-TP impedes the completion of the conformational helical changes within the four catalytic sites, which in turn blocks the dNTP hydrolysis activity of SAMHD1. Therefore, the Y374 may not act by a steric gate mechanism like L150 to exclude certain nucleotides from the catalytic pocket of SAMHD1.

### Ara-CTP does not fit into the A2 site of SAMHD1

**Fig 5:**
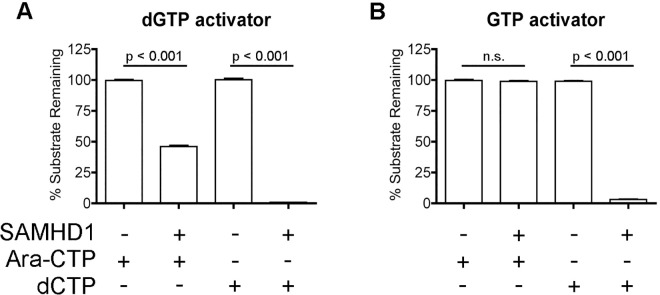
Ara-CTP does not fit into the A2 site of SAMHD1. A) Evaluating ara-CTP hydrolysis in the presence of dGTP, using as A1site activator. When dGTP was present, ara-CTP and dCTP were significantly hydrolyzed (p < 0.001) by SAMHD1. Data are presented as the percent compound remaining (y-axis). B) Determining if ara-CTP is hydrolysis by SAMHD1 in the presence of GTP. GTP will only fit into the A1 site of SAMHD1, thus requiring ara-CTP to occupy the A2 and catalytic sites for ara-CTP hydrolysis to occur. The percentage of ara-CTP remained constant with and without SAMHD1, indicating that ara-CTP cannot occupy the A2 site. Reactions containing dCTP was conducted and led to hydrolysis of dCTP in the presence of SAMHD1. Mean and SEM are plotted with significant or no significant (n.s.) differences determined using T test analysis.

We then investigated if ara-CTP could permit homotetramerization of SAMHD1 by entering the A2 site. In order to accomplish this, we simply compared ara-CTP degradation in the presence of dGTP or GTP plus SAMHD1 protein. According to the SAMHD1 model (Fig 1), GTP can only occupy the A1 site, and thus requiring ara-CTP to occupy both A2 sites and catalytic sites to have hydrolysis active. However, dGTP can occupy A1, A2 and catalytic sites to promote SAMHD1 homotetramerization and hydrolysis activity. As shown in Fig 5A, when dGTP is used as the A1 activator, ara-CTP as well as dCTP (control) were significantly hydrolyzed (p < 0.001) when SAMHD1 was present. However, ara-CTP was not hydrolyzed when GTP and SAMHD1 was present, indicating that ara-CTP cannot occupy the A2 site of SAMHD1 in order to allow homotetramerization. For the control reaction, dCTP was significantly hydrolyzed (p < 0.001) in the presence of SAMHD1 and GTP (Fig 5B).

### Determining the *Ki* of SMDU-TP

**Fig 6:**
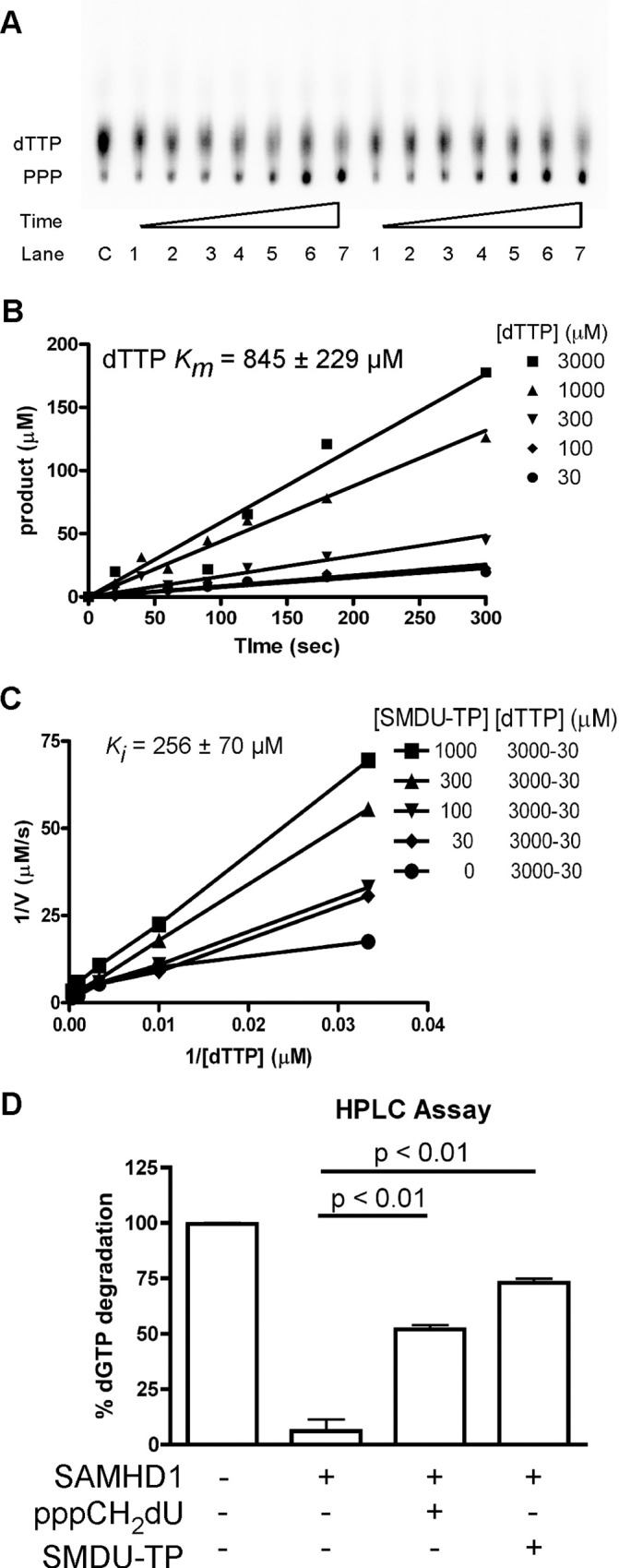
Biochemical assessing SMDU-TP. (A) Representative TLC plate showing ^32^-PPP accumulations over 20–300 sec. time course when using 1.25 μCi/μL [γ-^32^P]-dTTP with 3000 (left side) and 1000 (right side) μM of dTTP (cold) as the substrate in the presence of 1 μM of SAMHD1 enzyme. (B) Graphing data from TLC analysis to generate slopes for the different dTTP concentrations tested. Data displayed as Product (μM) (y-axis) *vs*. Time (sec) (x-axis). *K*_*m*_ of dTTP was calculated to be 845 ± 229 μM from the slopes generated using Prism software. (C) TLC analysis was done at various concentrations of dTTP (3000–30 μM) in the presence of various concentrations of SMDU-TP (1000–30 μM). Data are graphed as 1/V (μM/s) (y-axis) *vs*. 1/[dTTP] (μM) (x-axis). From the slopes generated, the *K*_*i*_ of SMDU-TP was calculated to be 256 ± 70 μM. (D) Biochemical HLPC analysis of reactions with and without SAMHD1, and in the presence of pppCH_2_dU or SMDU-TP analog is graphed. We observed that both pppCH_2_dU and SMDU-TP analogs inhibit SAMHD1’s activity ((p < 0.01) by one-way ANOVA analysis with Bonferroni’s multiple comparisons), leading to more dGTP substrate remaining after the 2 h incubation with enzyme. All data are representative of two independent studies with mean and SEM displayed.

Seamon *et al*. demonstrated that the pppCH_2_dU analog, a non-hydrolysable SAMHD1 inhibitor, fits into both A2 and catalytic sites of SAMHD1, leading to two different *Ki* values and mechanisms of SAMHD1 inhibition. Since the (2'*S*)-2'-OH sugar moiety (ara-CTP; Fig 5) cannot fit within the A2 site of SAMHD1, we speculate that SMDU-TP analog, which has a (2'*S*)-2'-methyl moiety, will also be excluded from the A2 site. Therefore, the SMDU-TP analog may only inhibit the dNTPase activity of SAMHD1 at the catalytic site. A modified TLC assay procedure was used to determine the *Km* of dTTP (substrate) and *Ki* of SMDU-TP analog [15]. A representative TLC gel is displayed for dTTP hydrolysis by SAMHD1, showing the accumulation of the ^32^-PPP product from [γ-^32^P]-dTTP over 20–300 s without inhibitor (Fig 6A). Control (C), having no SAMHD1 enzyme, is used to subtract out the ^32^-PPP background amount. Kinetic data are plotted and used to calculate the *Km* of dTTP to be 845 ± 229 μM (Fig 6B; dTTP only). Next SMDU-TP analog was evaluated at various concentrations: 1000–30 μM, in the presence of various concentrations of dTTP (3000–30 μM). These data were graphed in Fig 6C. The *Ki* for SMDU-TP analog was calculated to be 256 ± 70 μM under our experimental conditions. We evaluated 1 mM pppCH_2_dU analog or 1 mM SMDU-TP analog in the presence of 1 mM dGTP and found that both pppCH_2_dU and SMDU-TP analogs could inhibit the dNTP triphosphohydrolase activity of SAMHD1 (p < 0.01) under our experimental HPLC assay conditions (Fig 6D). However, neither pppCH_2_dU nor SMDU-TP analog could completely abolish the dNTP triphosphohydrolase activity of SAMHD1 in the presence of canonical dGTP in this assay. Overall, the SMDU-TP analog appears to be a competitive inhibitor of SAMHD1.

### Monitoring catabolism of ara-CTP by SAMHD1 in monocyte-derived macrophages

**Fig 7:**
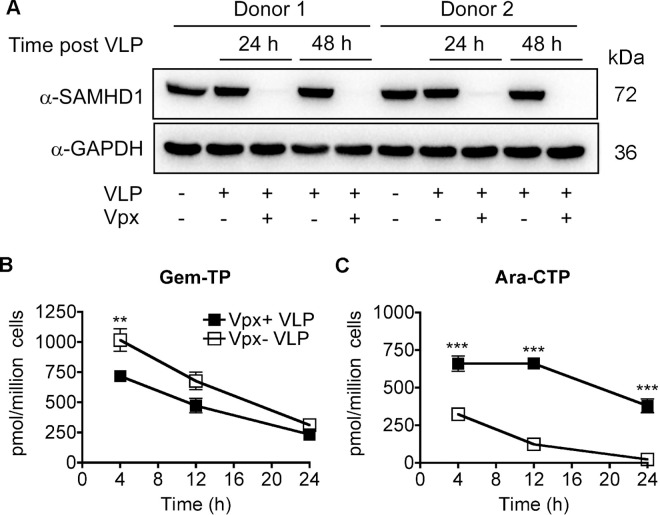
Monitoring ara-CTP and gem-TP concentrations in MDMs. A) Monocyte-derived macrophages (MDMs) were pretreated with virus-like particles (VLP) one day prior to replacing the medium with fresh medium plus compounds: 10 μM of ara-C (cytaribine-^13^C_3_) or 10 μM of gemcitabine. Whole cell lysates were collected at 0, 24 and 48 h post VLP addition. Lysates were analyzed by immunoblotting for SAMHD1 and GAPDH, loading control. SAMHD1 protein levels were reduced at 24 h after Vpx+ VLP exposure. Two human primary MDM donors are shown. Cellular nucleotide extracts were generated at 4, 12 and 24 h post drug addition from treated MDMs. The intracellular concentrations of B) gem-TP (2',2'-diF-dCTP) and (C) ara-CTP were quantified from the extracts using HPLC-MS/MS analysis. Data are plotted as pmol/million cells (y-axis) *vs*. time (h) (x-axis). Gem-TP is a significantly lower (**, p < 0.01; T test) in the Vpx+ VLP treated MDMs at 4 h after drug addition. However, the rate of gem-TP decay is comparable between the two groups, suggesting that gem-TP degradation is SAMHD1 independent. Ara-CTP concentrations are significantly higher (***, p < 0.001) at 4, 12 and 24 h for the Vpx+ VLP treated MDMs. Moreover, the rate of decay of ara-CTP is slower in Vpx+ VLP treated group, suggesting ara-CTP turnover is SAMHD1 dependent. Data are from two independent donors tested in duplicate.

To extend our biochemical data confirming that ara-CTP (Fig 2B) is a substrate for SAMHD1, we used a well-defined tissue culture model of monocyte-derived macrophages (MDMs) treated with virus-like particles (VLP) [33, 45, 46] to evaluate changes in ara-CTP concentration in the absence of SAMHD1 *in vivo*. When MDMs are treated with VLP containing SIV_mac239_ viral protein X (Vpx), a rapid decrease in SAMHD1 protein level that last for several days after Vpx+ VLP exposure [45, 46]. MDMs were exposed to VLP with and without Vpx for 24 h before the medium was replaced with fresh medium containing 10 μM of ara-C or 10 μM of gemcitabine. Cell lysates were collected at 24 and 48 h post VLP addition to monitor SAMHD1 protein level. Immunoblots show the depletion of SAMHD1 in MDMs treated with Vpx+ VLP, but not Vpx- VLP treated MDMs and control MDMs (no VLP treatment) (Fig 7A).

Next, cellular dNTP extracts were collected at 4, 12 and 24 h post medium change with drug. HLPC-MS/MS analysis was used to quantify the intracellular concentrations of gem-TP (2',2'-diF-dCTP) and ara-CTP. We found that the cellular gem-TP concentration at 4 h is significantly lower (**, p <0.01) in Vpx+ VLP treated MDMs as compared to Vpx- VLP MDMs (Fig 7B). This could be due to changes in of cellular kinase activities that phosphorylate nucleosides. Deoxycytidine kinase, which phosphorylates dC to dCMP, is negatively regulated by dCTP, the reaction pathway end product [47]. Importantly we see a comparable rate decrease in gem-TP concentration between the two treatment groups from 4 to 24 h (Fig 7B), suggesting gem-TP turnover was SAMHD1 independent. Next, we evaluated ara-C treatment in the two MDM populations (Fig 7C). The Vpx+ VLP treated MDMs had significantly (***, p <0.001) higher levels of ara-CTP at 4, 12 and 24 h, as compared to Vpx- VLP treated MDMs, suggesting SAMHD1 impacts the peak intracellular ara-CTP concentration and suggest augmentation of ara-CTP turnover rate *in vivo*. Our tissue culture findings support our biochemical studies strongly suggesting that ara-CTP may be a substrate for SAMHD1 *in vivo*. Moreover, our data reveals that an additional cellular pathway, SAMHD1 independent, is present that is involved with the turn over gem-TP in the cell, which requires additional studies to elucidate the mechanism in the future.

## Discussion

**Fig 8:**
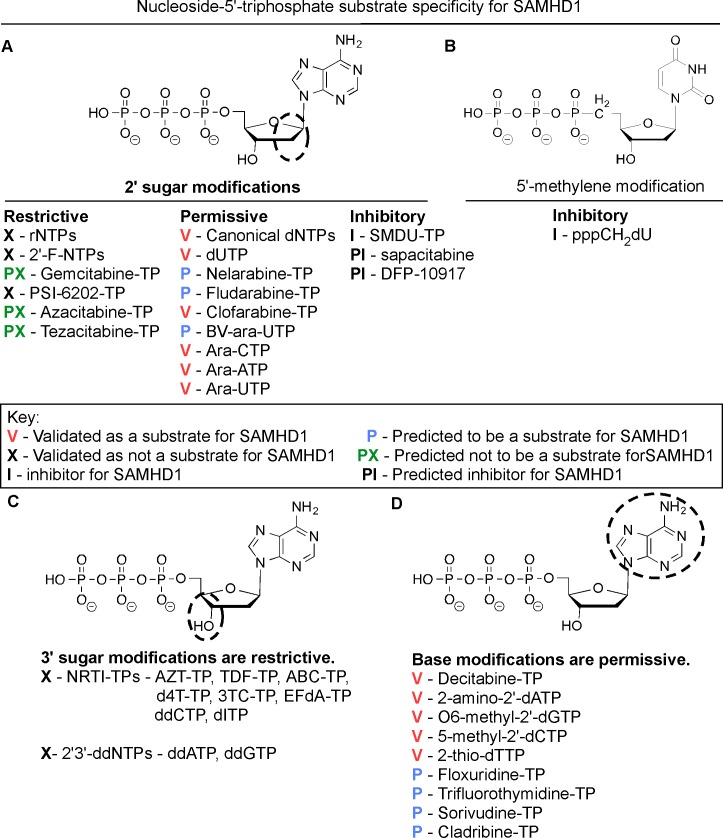
Determining nucleotide analog specificity for SAMHD1. A) Modification of the 2' sugar position of a nucleotide can lead to several different outcomes. First, (2'*R*)-2'-F and (2'*R*)-2'-OH sugar moieties have been shown not to be substrates for SAMHD1. Additional analogs with (2'*R*)-2'-F and (2'*R*)-2'-OH sugar moieties would be predicted not to be substrates for SAMHD1. Second, canonical dNTPs and the non-canonical dUTP are substrates for SAMHD1. Our data shows that ((2'*S*)-2'-OH) arabinose nucleoside-5'-triphosphates are also substrates for SAMHD1. Therefore, we also predict several other arabinose nucleoside analogs would be substrates for SAMHD1. Moreover, clofarabine-TP ((2'*S*)-2'-F) was reported hydrolyzed by SAMHD1 [43]. Finally, we found the SMDU-TP, (2'*R*)-2'-methyl sugar moiety, inhibited the triphosphohydrolase activity of SAMHD1. We postulate that the (2'*R*)-2'-methyl moiety may prevent the conformational change in the catalytic site of SAMHD1 due to the size of the methyl group clashing with Y374. Therefore, we predicted that nucleotides with a (2'*S*)-2'-cyano moiety may also inhibit dNTPase activity of SAMHD1. B) A SAMHD1 inhibitor has been reported [34]. The pppCH_2_-dU analog has a 5'-methylene modification, making the analog non-hydrolysable in the catalytic site, but also was shown to block homotetramerization when present in the A2 site [34]. C) Modification of the 3'-OH sugar moiety is not permissive. NRTIs and ddNTPs lack a 3'-OH moiety, making them chain terminators for DNA polymerases, are not substrates for SAMHD1. D) Base modifications for different nucleoside analogs are permissive substrates for SAMHD1.

In this study we explore the effect of stereoselective 2' sugar moiety substitution analogs on the dNTPase activity of SAMHD1. In Fig 8, we compile our results for what we know will influence nucleotides to be substrates for SAMHD1. Ji *et al*. proposed that L150 and Y374 of SAMHD1 form a tight catalytic pocket to exclude rNTPs. The L150 would essentially act as a steric gate to exclude rNTPs from the catalytic pocket (Fig 8A). Our computational modeling indicates that only a dNTP can fit in the catalytic site (Fig 2A), but larger (2’*R*)-2’-F or (2’*R*)-2’-OH moieties are excluded (Fig 2C and 2E). A HLPC-bases assay confirmed that (2'*R*)-2'-F and (2'*R*)-2'-OH are not substrates for SAMHD1 (Fig 2D, 2F and S1 Fig). Therefore, the L150 acts by a steric gate mechanism, i.e. clashing of the L150 with the (2'*R*)-2'-F/OH moiety to exclude these nucleotides from docking in the catalytic pocket of SAMHD1. Our computational modeling indicates that (2'*S*)-2'-H and (2'*S*)-2'-OH sugar moieties can fit within the catalytic site of SAMHD1, while (2'*S*)-2'-methyl moiety clash with Y374 (Fig 4). Biochemical analysis showed that both dCTP ((2'*S*)-2'-H) and ara-CTP ((2'*S*)-2'-OH) are hydrolyzed in the presence of SAMHD1 (Fig 4B and 4D). We therefore postulate that ara-CTP, ara-ATP, ara-GTP, fludarabine-TP, cladribine-TP, and clofarabine-TP would be sensitive to hydrolysis by SAMHD1 *in vivo* (Fig 8A). Interestingly, the SMDU-TP analog ((2'*S*)-2'-methyl) blocked the triphosphohydrolase activity of SAMHD1 in the biochemical assay, making it a nucleotide inhibitor of SAMHD1. To address why SMDU-TP inhibits the dNTPase activity of SAMHD1, we postulate that SMDU-TP prevents the full A351-V378 helix 10Å movement towards a dNTP substrate in the catalytic pocket [5]. This mechanism is very different from the SAMHD1 inhibitor, pppCH_2_dU (Fig 8B), which acts by preventing tetramer formation and is a non-cleavable substrate for SAMHD1 [34]. This means that (2'*S*)-2' substituted nucleotides has access to and bind within the catalytic pocket of SAMHD1. For our mechanism to work, the final changes with the A351-V378 helix movement is completed after the dNTP is docket within the catalytic site. Once the helix conformational change is completed then hydrolysis of the nucleotide analog occurs. For SMDU-TP, the (2'*S*)-2'-methyl clashes with Y374 preventing the helix from completing the conformational change and thus blocks hydrolysis. If our model is correct, then we would predict that sapacitabine (CYC682) and DFP-10917, which have a (2'*S*)-2'-cyano (CN) moiety, would also dock within the catalytic pocket of SAMHD1 and then inhibit the dNTPase activity of SAMHD1 (Fig 8A). Sapacitabine and DFP-10917 are currently under clinical investigation as anticancer nucleoside compounds.

Additional aspects of nucleoside analogs are also examined. The 3' position of the sugar is of high importance in determining which deoxynucleoside-5'-triphosphate analogs have the potential to be substrates for SAMHD1 (Fig 8C). Ji *et al*. proposed that the 3'-OH sugar moiety has hydrogen bond interactions with D319 and Q149 of SAMHD1 to promote correct alignment of the dNTP in the catalytic pocket [31]. The FDA-approved antiviral NRTIs, such as zidovudine (AZT), stavudine (d4T), lamivudine (3TC), zalcaiabine (ddC; Fig 3C), didanosine (ddI; Fig 3D), and abacavir (ABC) were shown not to be substrates for SAMHD1 *in vitro* (this study and [17, 18]). Additionally, we show that ddATP and ddGTP are not substrates for SAMHD1 (Fig 3A and 3B). Collectively these data validate Ji *et al*. biochemical structure model, indicating that 3'-OH sugar moiety is an essential function group of the nucleoside for SAMHD1 substrate specificity [31]. As indicated in Fig 8D, the type of base or being modified does not restrict a nucleotide analog from being a substrate for SAMHD1. Presently, all canonical bases and modified bases analogs: decitabine-TP (S1 Fig), dUTP, 2-amino-2'-dATP, O6-methyl-2'-dGTP, 5-methyl-2'-dCTP and 2-thio-dTTP are hydrolyzed by SAMHD1 [34, 40]. Therefore, we postulate the following non-canonical FDA-approved nucleosides: cladribine, floxuridine, trifluorothymidine, and sorivudine, when phosphorylated in the cell, are strong candidates for being SAMHD1 substrates *in vivo*. These are nucleosides used for anticancer and antiviral treatments.

Biologically, both ribonucleotide reductase and SAMHD1 have roles in maintaining proper intracellular dNTP concentrations [48–50]. SAMHD1-deficient mice have a dNTP imbalance, with higher intracellular dATP and dGTP concentrations than dTTP and dCTP concentrations [51], and a cellular dNTP imbalance can promote higher rates of mutagenesis in cancer cells, and influence the ability of DNA viruses to infect cells [33, 52–55]. One can imagine that a SAMHD1 nucleoside analog inhibitor might be useful in an anticancer regiment by promoting a dNTP imbalance in rapidly dividing cancer cells or in combination therapy with FDA-approved nucleoside analogs that are sensitive to hydrolysis by SAMHD1. Alternatively, increasing SAMHD1 levels in cancer cells may slow cell growth by decreasing dNTP concentrations [56]. Overall our data provides insights as to how stereoselective 2' sugar moiety substitutions impact the triphosphohydrolase activity of SAMHD1.

## Supporting Information

S1 Fig Evaluating additional nucleotide as substrates for SAMHD1. A and B) canonical dATP and dTTP were analyzed. Using semi-quantitative HLPC analysis method, compounds were incubated with and without 1.6 μM of SAMHD1 enzyme plus dGTP (A1 site activator) to determine if they are substrates of SAMHD1. dATP, dTTP and dGTP are significantly (p < 0.001) hydrolyzed in the presence of SAMHD1. C) Decitabine-5'-triphosphate (decitabine-TP) is a base modified nucleotide. Decitabine-TP and dGTP are significantly (p < 0.001) hydrolyzed in the presence of SAMHD1. D) (2'*R*)-2'-F-dATP was not hydrolyzed for SAMHD1 *in vitro*, whereas the internal dGTP control in the same reaction was significantly (p < 0.001) hydrolyzed in the presence of SAMHD1. E-G) Ribonucleotide-5'-triphosphates: ATP, GTP and UTP are evaluated in the biochemical assay and were not significantly hydrolyzed by SAMHD1. Mean and SEM are plotted with significant or no significant (n.s.) differences determined using T test analysis.
(TIF)

S2 Fig Site-directed mutagenesis for L150 and Y374. A and B) Site-directed mutagenesis was used to make (A) L150V and (B) L150A mutants of SMAHD1. Biochemical analysis shows that both L150 mutants fail to significantly (n.s.) hydrolyzed dGTP. C-E) Y374 mutants: Y374F, Y374I, and Y374A were generated by site-directed mutagenesis. Biochemical analysis shows that both L150 mutants did not hydrolyze dGTP over the 2 hour incubation period. Mean and SEM are plotted with no significant (n.s.) differences determined using T test analysis.
(TIF)

S3 Fig Evaluating additional non-canonical nucleotides as substrates for SAMHD1. A and B) Arabinose nucleotides: ara-ATP and ara-UTP were evaluated in the presence of dGTP using a semi-quantitative HLPC analysis method. Compounds were incubated with and without 1.6 μM of SAMHD1 enzyme plus dGTP (A1 site activator) to determine if they are substrates of SAMHD1. Ara-ATP, Ara-UTP and dGTP are significantly (p < 0.001) hydrolyzed in the presence of SAMHD1. C) SMDU-TP ((2'*S*)-2'-methyl-dUTP) appears to block dGTP hydrolysis when at 1 mM for each nucleotide in the reaction tube. Therefore, 0.1 mM SMDU-TP and 1 mM dGTP are incubated for 2 h in the presence or absence of SAMHD1. No significant (n.s.) decrease in the percent SMDU-TP was detected, whereas dGTP was significantly (p < 0.001) hydrolyzed by SAMHD1. D) PSI-6296-TP has a (2'*R*)-2'-F, (2'*S*)-2'-methyl-dUTP. PSI-6202-TP is not hydrolyzed by SAMHD1, whereas dGTP, which is in the same reaction, is significantly (p < 0.001) hydrolyzed by SAMHD1.
(TIF)
