# Supplementary figures and images for: Substrates and Inhibitors of SAMHD1
Source: PLoS One. 2017 Jan 3;12(1):e0169052. doi: 10.1371/journal.pone.0169052 (PMC5207538; doi:10.1371/journal.pone.0169052)

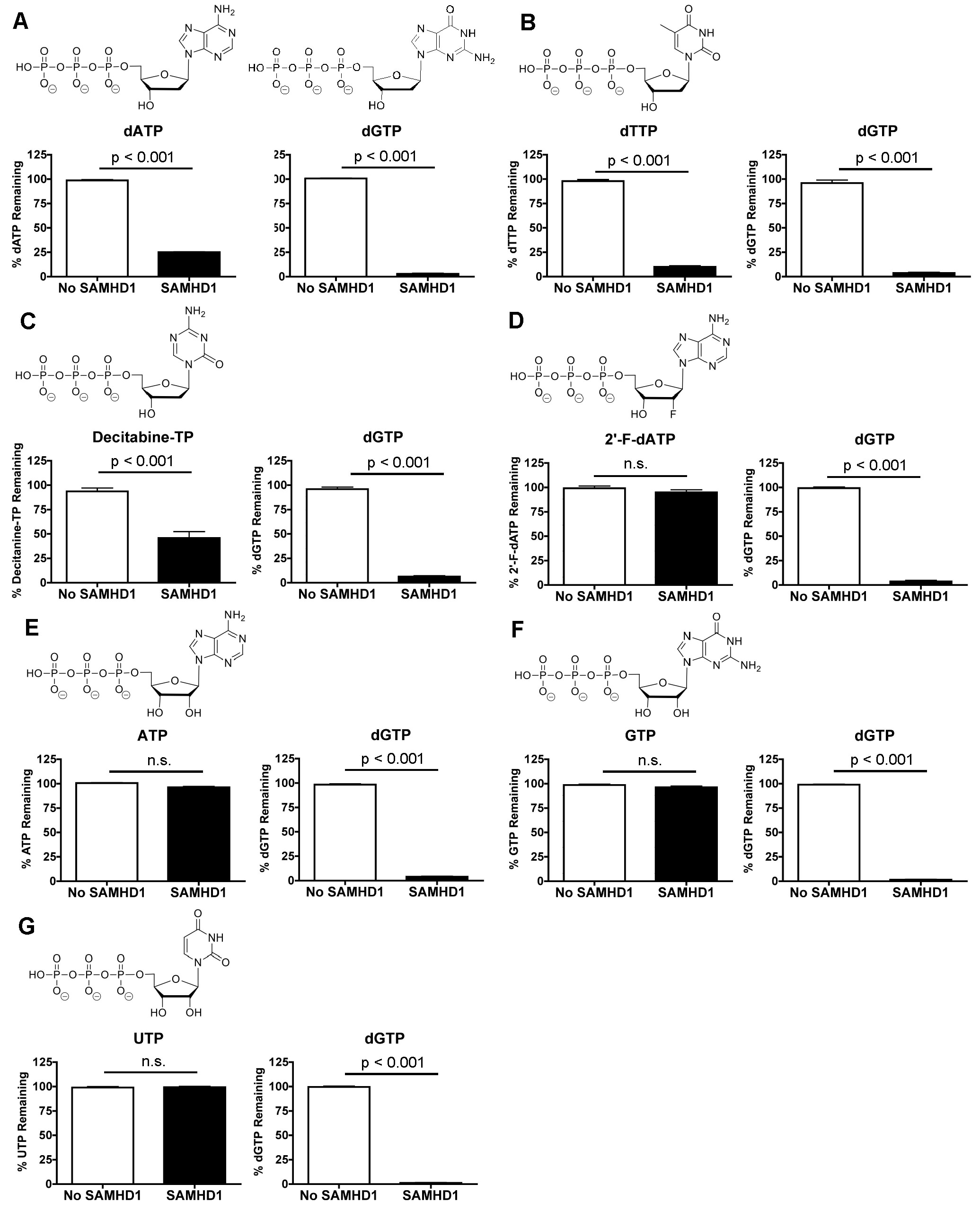

Supplement: S1 Fig — A and B) canonical dATP and dTTP were analyzed. Using semi-quantitative HLPC analysis method, compounds were incubated with and without 1.6 μM of SAMHD1 enzyme plus dGTP (A1 site activator) to determine if they are substrates of SAMHD1. dATP, dTTP and dGTP are significantly (p < 0.001) hydrolyzed in the presence of SAMHD1. C) Decitabine-5'-triphosphate (decitabine-TP) is a base modified nucleotide. Decitabine-TP and dGTP are significantly (p < 0.001) hydrolyzed in the presence of SAMHD1. D) (2'R)-2'-F-dATP was not hydrolyzed for SAMHD1 in vitro, whereas the internal dGTP control in the same reaction was significantly (p < 0.001) hydrolyzed in the presence of SAMHD1. E-G) Ribonucleotide-5'-triphosphates: ATP, GTP and UTP are evaluated in the biochemical assay and were not significantly hydrolyzed by SAMHD1. Mean and SEM are plotted with significant or no significant (n.s.) differences determined using T test analysis. (TIF) [file pone.0169052.s001.tif]

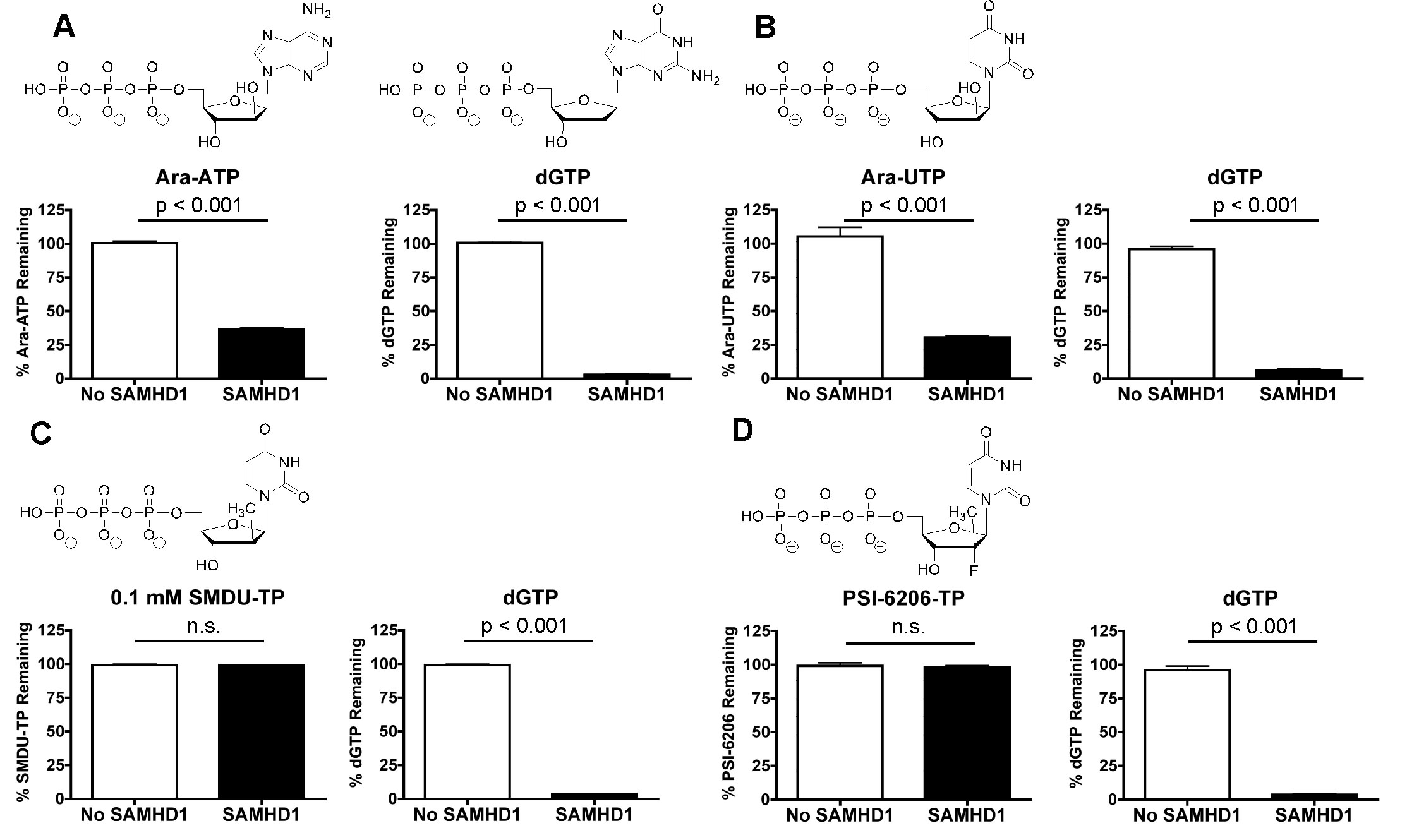

Supplement: S3 Fig — A and B) Arabinose nucleotides: ara-ATP and ara-UTP were evaluated in the presence of dGTP using a semi-quantitative HLPC analysis method. Compounds were incubated with and without 1.6 μM of SAMHD1 enzyme plus dGTP (A1 site activator) to determine if they are substrates of SAMHD1. Ara-ATP, Ara-UTP and dGTP are significantly (p < 0.001) hydrolyzed in the presence of SAMHD1. C) SMDU-TP ((2'S)-2'-methyl-dUTP) appears to block dGTP hydrolysis when at 1 mM for each nucleotide in the reaction tube. Therefore, 0.1 mM SMDU-TP and 1 mM dGTP are incubated for 2 h in the presence or absence of SAMHD1. No significant (n.s.) decrease in the percent SMDU-TP was detected, whereas dGTP was significantly (p < 0.001) hydrolyzed by SAMHD1. D) PSI-6296-TP has a (2'R)-2'-F, (2'S)-2'-methyl-dUTP. PSI-6202-TP is not hydrolyzed by SAMHD1, whereas dGTP, which is in the same reaction, is significantly (p < 0.001) hydrolyzed by SAMHD1. (TIF) [file pone.0169052.s003.tif]
